# Mantle flow and deep electrical anisotropy in a main gateway: MT study in Tierra del Fuego

**DOI:** 10.1038/s41598-019-43763-w

**Published:** 2019-05-09

**Authors:** Lourdes González-Castillo, Fernando Bohoyo, Andreas Junge, Jesús Galindo-Zaldívar, Marcel Cembrowski, Pablo Torres-Carbonell, Ana Ruiz-Constán, Antonio Pedrera, Pedro Ibarra, Adolfo Maestro, Patricia Ruano

**Affiliations:** 1 Departamento de Geodinámica, Universidad de Granada, Granada, Spain; 2 Instituto Geológico y Minero de España (IGME), Madrid, Spain; 3 Goethe-Universität Frankfurt am Main, Frankfurt, Germany; 4 IACT, CSIC-Universidad de Granada, Granada, Spain; 5 Centro Austral de Investigaciones Científicas (CONICET), Ushuaia, Argentina

**Keywords:** Geodynamics, Geophysics

## Abstract

Asthenospheric mantle flow drives lithospheric plate motion and constitutes a relevant feature of Earth gateways. It most likely influences the spatial pattern of seismic velocity and deep electrical anisotropies. The Drake Passage is a main gateway in the global pattern of mantle flow. The separation of the South American and Antarctic plates since the Oligocene produced this oceanic and mantle gateway connecting the Pacific and Atlantic oceans. Here we analyze the deep crustal and upper mantle electrical anisotropy of its northern margin using long period magnetotelluric data from Tierra del Fuego (Argentina). The influence of the surrounding oceans was taken into account to constrain the mantle electrical conductivity features. 3D electrical models were calculated to fit 18 sites responses in this area. The phase tensor pattern for the longest periods reveals the existence of a well-defined NW-SE electrical conductivity anisotropy in the upper mantle. This anisotropy would result from the mantle flow related to the 30 to 6 Ma West Scotia spreading, constricted by the subducted slab orientation of the Pacific plate, rather than the later eastward mantle flow across the Drake Passage. Deep electrical anisotropy proves to be a key tool for a better understanding of mantle flow.

## Introduction

**Figure 1:**
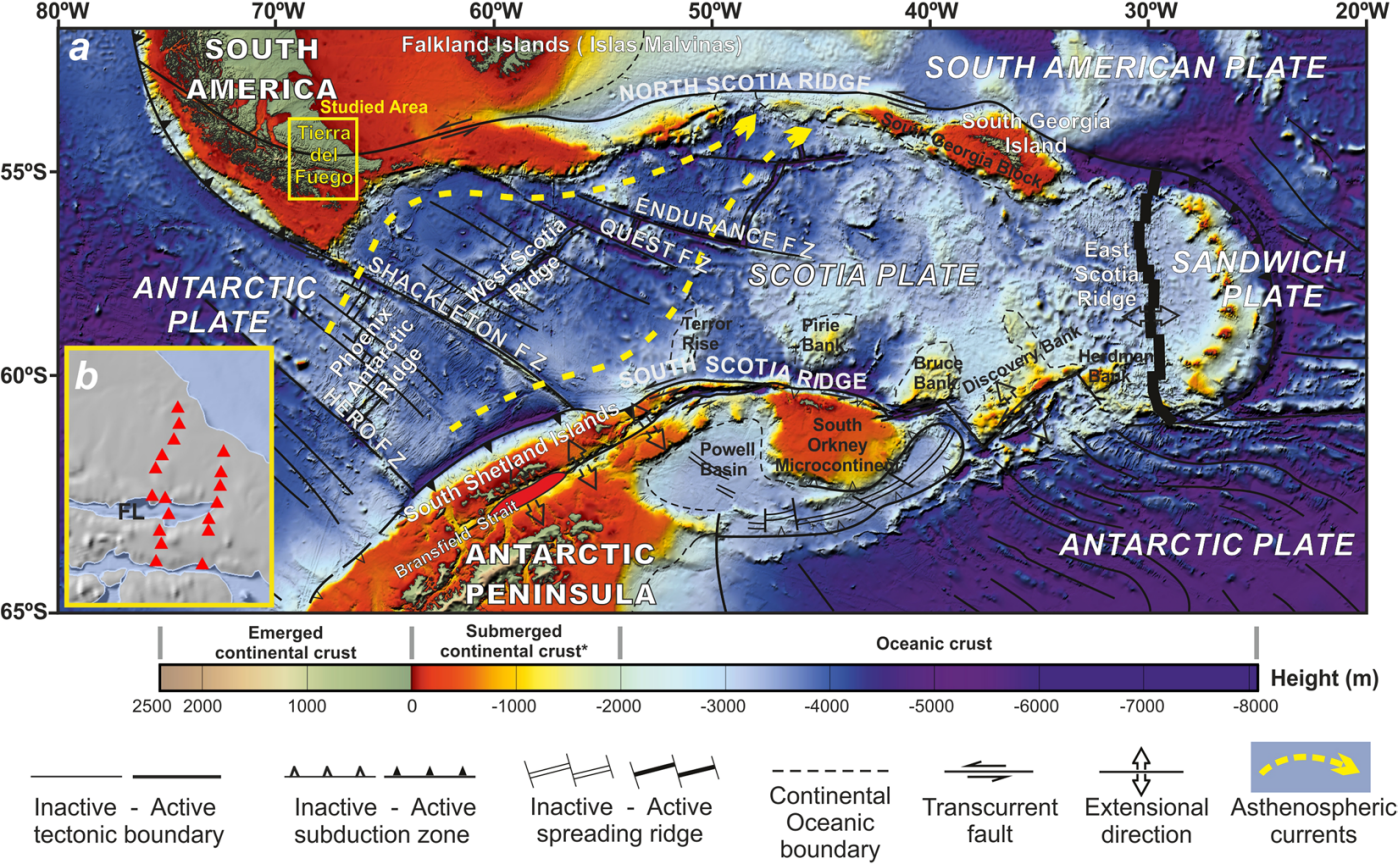
Tectonic structure of the Scotia Arc. (**a**) Location of the studied area is marked by yellow square. (**b**) Map view including sites position (red triangles); FL, Fagnano Lake. Generated from GlobalMapper 17 http://www.bluemarblegeo.com/products/global-mapper.php and CorelDraw x8 https://www.coreldraw.com.

The Earth’s evolution is guided by continental drift, ultimately driven by mantle flows^1^. The understanding of mantle convection and hence, the geodynamic processes, strongly depends on the behaviour of the Lithosphere-Astenosphere boundary (LAB)^2,3^. The LAB has been estimated to rapidly increase in oceanic floors younger than 55 Ma and to be deeper or equal to 150 km in the transition between oceanic-continental lithosphere^2^. Whereas subduction zones represent major mantle flow barriers, areas of relatively weak continental lithosphere may connect different oceans along mantle gateways^4^. Mantle cells related to seafloor spreading represent a suitable process provoking mantle flow and the subsequent anisotropy orthogonal to the spreading axis. Thus, in recent structures without overprinted deformations, mantle fabric consistently fits paleo-spreading directions in oceanic basins^5^. Seismic anisotropy studies provide widespread results for a broad depth range, as well as more detailed data restricted to the mantle top^6^. In contrast, long period magnetotelluric (LMT) research allows for a comparatively high depth resolution of mantle electrical anisotropy^7–11^. Apart from mineralogical composition, laboratory studies reveal the temperature and pressure dependence of the electrical conductivity of the rocks. Additionally, water conditions, partial melt as well as oxygen or hydrogen fugacity influence the electrical features^8^. Several investigations have related crustal electrical conductivity with the presence of saline fluids or highly conductive minerals (eg; graphite, sulphides or Fe/Ti oxides)^12–14^. Whereas the electrical properties of the upper mantle are constrained to temperature dependence in case of dry olivine, partial melting or presence of water, the asthenopheric mantle electrical anisotropy is not accurately constricted. Given the homogeneity of asthenospheric mantle composition, the origin of its electrical conductivity and anisotropy is proposed to be a consequence of interstitial melts and/or the olivine elongation. Mantle flow determines the elongation of the olivine crystals which constrain the shape of interstitial space occupied by melts and hence the electrical anisotropy direction. The strain in olivine due to mantle flow favours pencil glide [1 0 0]-^11,15–17^. Hydrogen diffusion along these olivine elongation axis, explains the existence of preferential conductivity directions in context with a very low fraction of partial melt^7,8,11,18^. Electrical anisotropy has been used in a number of geological contexts to date^7–11,14^, but never with a focus on mantle gateways. The Drake Passage (Fig. 1) is a major mantle and oceanic gateway driving the global pattern of the Earth´s mantle flow^19^. It was formed since the Oligocene through the fragmentation of the prior continental connection between South America and the Antarctic Peninsula, and the subsequent development of the Scotia Arc^19^. The Arc itself is composed by the Scotia and Sandwich plates, in between the South American and Antarctic plates. Tierra del Fuego (South America), located in the continental lithospheric area at the northern branch of the Scotia Arc, is crossed by the South America-Scotia left-lateral transcurrent plate boundary. This area, at the northern margin of the mantle gateway, draws particular interest in terms of the effect of mantle flow. Petrological^20^ and geophysical data —including gravity research^21^— provide evidence supporting the inception of the Pacific mantle in the Atlantic during the development of the tectonic arc. However, other studies hold the absence of mantle flow from Pacific in this gateway in view of petrological data and the topography evolution of the Scotia Sea during the development of the Scotia Arc^22,23^. Anyway, the Scotia Arc deep structure is poorly known due to the scarce distribution of seismic stations located in its eastern and southern parts^24,25^.

## Methodology and Results

**Figure 2:**
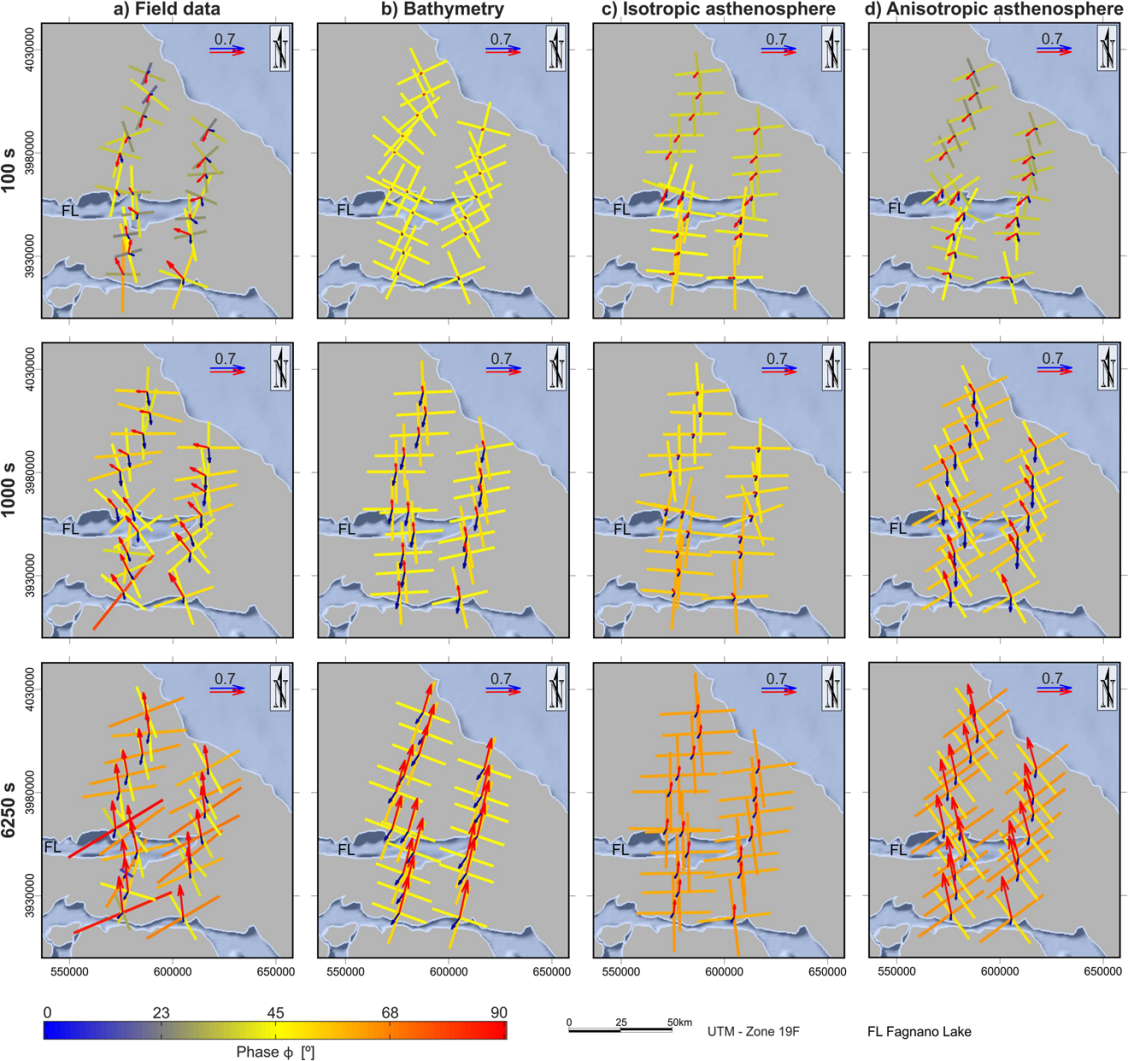
Phase tensor, real part (red) and imaginary part (blue) of tipper (Wiese convention) for all field sites. (**a**) Observations. (**b**) 3D bathymetry model. (**c**) 3D model considering bathymetry, the main geological units and an isotropic asthenosphere. (**d**) 3D model considering bathymetry, the main geological units and an anisotropic asthenosphere. Modeling results are compared for the periods 100 s, 1000 s and 6250 s. FL, Fagnano Lake. Structures details in Fig. 3.

In 2012, a long period magnetotelluric (LMT) study with 18 stations distributed along two profiles was performed in Tierra del Fuego to investigate the deep crustal and upper mantle conductivity (Fig. 1b). The three magnetic and two telluric field components were recorded at a sampling rate of 4 Hz using a LEMI-417 system. Standard robust processing yielded high quality transfer functions. Figure 2a displays the pattern of the phase tensor and the real and imaginary part of the tipper vector for the periods 100 s, 1000 s and 6250 s at the field sites. In the shorter period (100 s), there is a smooth transition between the phase tensor for the northern and southern sites. Phases beneath 45° indicate an increase of resistivity with depth toward the north. Tipper orientations abruptly change for sites north and south of Fagnano Lake. With increasing period, tipper vectors reveal a homogeneous pattern rotating clockwise from pointing to NW at 1000 s (average −47° ± 17°) to almost pointing to N at 6250 s (average −8° ± 6°). It is noteworthy that the magnitude of the tippers decreases towards the North as the distance increase from the Drake Passage. Phase tensor values increase with period, with a remarkable homogeneous split of about 20° at 6250 s. Thereby the 45° principal axes take almost the same direction as the tipper vectors with 65° phases for the orthogonal axis. Thus, phase tensors reveal a NW-SE conductive direction at depth.

**Figure 3:**
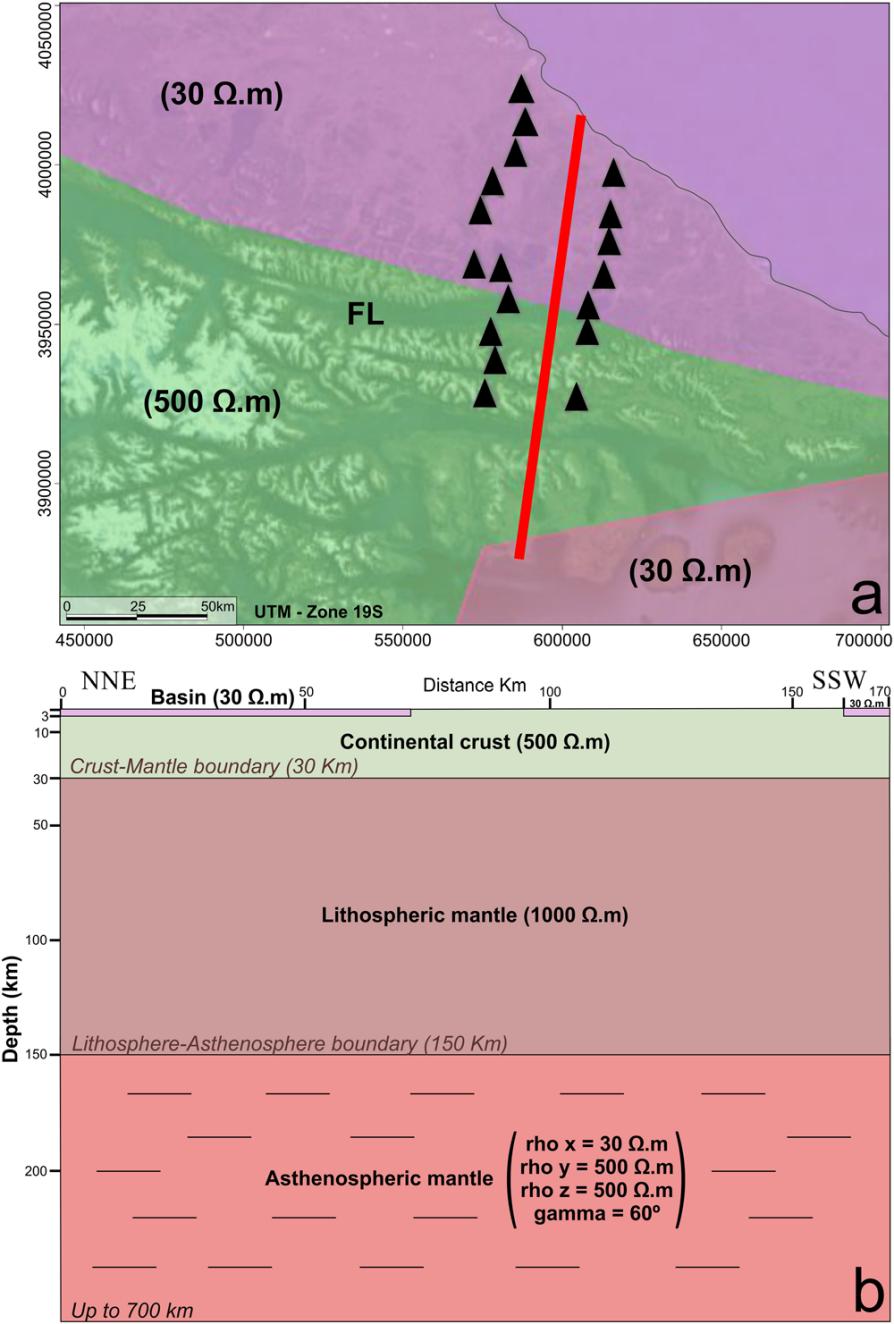
Main geological structures considered for 3D models. (**a**) Map view. FL, Fagnano Lake. (**b**) Idealized cross section for model with anisotropic astenosphere. Astenosphere resistivity for isotropic model (30 Ωm). Background resistivity 500 Ωm. Sea water resistivity 0.25 Ωm.

The influence of seawater on MT parameters (phase tensor and tipper vectors) at coastal areas strongly depends on the bathymetry^8,26,27^. Finite element (FE) models^26^ (Comsol Multiphysics 5.2TM, http://www.COMSOL.com/) were calculated to investigate seawater influence on the MT results in the study area (Fig. 2b) in view of GEBCO bathymetry (www.gebco.net) with an average seawater resistivity of 0.25 Ωm. The depth of investigation (skin depth) depends on period and resistivity (δ = 500 √ρT) where δ has units of meters, T is the period in seconds and ρ is the electrical resistivity in Ωm^28^. Figure 3 covers the depth range found to be sensitive to our observations. The model size was frequency dependent and of the order of several skin depths with increasing mesh size from the center, thus providing sufficient resolution in the survey area and avoiding boundary effects. About 300.000 mesh elements roughly yielded 4 million degrees of freedom. A preliminary conductivity model reflects the main geological units, i.e. the conductive basins and the resistive lithosphere (Fig. 3). Furthermore, the influence of an isotropic (Fig. 2c) and an anisotropic (Fig. 2d) asthenosphere was considered. It is obvious that toward the end of the long period the conducting isotropic asthenosphere reduces the length of the tipper vectors considerably, while the phase split does not appear, either with or without asthenosphere. Additionally, the observed tipper vectors are rotated anti-clockwise by roughly 30° on average. We found that an anisotropic asthenosphere with a resistive principal axis in a N60°E direction (resp. the conductive principal axis in N150°E direction) explains both the large observed tipper vectors and the phase split along the two profiles (Fig. 2d).

## Discussion

**Figure 4:**
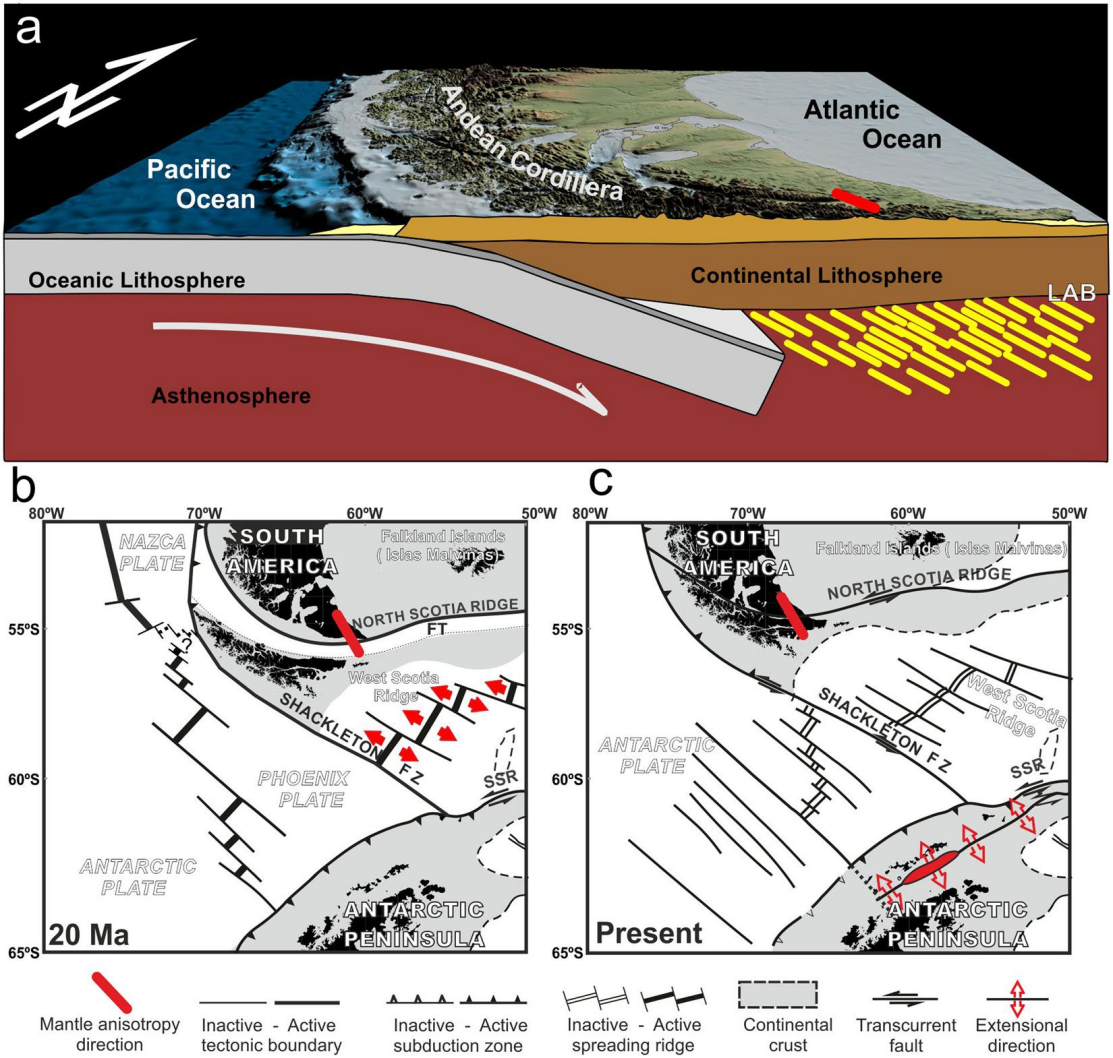
Geodynamic model for the northern branch of the Scotia Arc. (**a**) Oceanic spreading of the West Scotia Ridge conditioning the mantle electrical anisotropy. LAB, Lithosphere-Asthenosphere boundary. (**b**) Tectonic setting of the west Scotia Arc 20 Ma ago. (**c**) Present tectonic setting of the west Scotia Arc.

In summary, the bathymetry crucially influences the behavior of MT response functions at sites onshore. It is essential to account for bathymetry in any MT study in the Tierra del Fuego region. The implementation of an azimuthally anisotropic upper mantle improves the data fit significantly. The high conducting principle axis describes the assumed direction of mantle flow, confirming the importance of electrical mantle signatures reported in MT investigations. The main results of MT research in Tierra del Fuego reveal the existence of a main NW-SE oriented anisotropy in the upper mantle. Alternative models may be proposed to explain the mantle anisotropy in the northern Scotia Arc. We might consider former mantle anisotropies parallel to the Andes Cordillera, which has a general N-S trend along the South American continental margin and is arched up to an E-W trend in the southernmost part of the continent. However, such behavior would produce an ESE-WNW to E-W anisotropy parallel to the strike of the main geological structures, which is oblique to the N150°E conductive direction obtained in the region. Given this setting, we favor that the anisotropy may be related to the NW-SE oceanic spreading of the West Scotia Ridge (Fig. 4), active from 30 to 6 Ma^29^. Since the continental crustal rifting, the Drake Passage opening was driven by mantle flow linked to this oceanic spreading axis. Peridotite strain may evidence features of mantle flow that can be actual or remained. Nowadays, the anisotropy beneath Tierra del Fuego corresponds to the last strong flow recorded in that sector. Since the West Scotia Ridge was inactive, mantle flow was probably forced below the continental margin and confined to the west by the active Pacific subducted slab that may act as a barrier, blocking any mantle flow from the Pacific in this area^4^. In addition, there has not been time enough to significantly modify this recent anisotropy related to the spreading by any later geodynamic process. The N150°E frozen-in olivine fabric record the spreading direction, which is orthogonal to the West Scotia Ridge and began near the oceanic-continental lithospheric boundary. The possible development of NE-SW asthenospheric channels in the Drake Passage gateway^21^, besides other geodynamic processes related to the Scotia Arc development^19^, are not recorded in the mantle fabric beneath Tierra del Fuego. The new MT data thus help evidence a discrepancy between deep electrical anisotropy and shallow geological trends, and enhance our knowledge on the kinematics of a main gateway opening.
